# Characterization of skin function associated with obesity and specific correlation to local/systemic parameters in American women

**DOI:** 10.1186/s12944-017-0608-1

**Published:** 2017-11-13

**Authors:** Shinobu Mori, Akiko Shiraishi, Karen Epplen, Desiree Butcher, Daiki Murase, Yuka Yasuda, Takatoshi Murase

**Affiliations:** 1 Biological Science Laboratories, Kao Corporation, 2606 Akabane, Ichikai-machi, Haga-gun, Tochigi, 321-3497 Japan; 2 Spring Grove Laboratories, 375 Thomas More Parkway, Suite 112, Crestview Hills, KY 41017 USA; 3 Analysis Science Laboratories, Kao Corporation, 2606 Akabane, Ichikai-machi, Haga-gun, Tochigi, 321-3497 Japan

**Keywords:** Obesity, Skin function, Insulin resistance, Inflammation

## Abstract

**Background:**

Obesity is considered problematic not only as a major cause of diabetes, hypertension, and dyslipidemia, but also as a risk of intractable dermatosis; however influence of obesity on skin function has not been clarified. To clarify the mechanism of obesity-associated skin disorders, we aimed to characterize the skin function of subjects with obesity, and identify possible influencing factors.

**Methods:**

Complex analyses including instrumental measurement, biochemical and lipidomics were performed for facial skin and physical evaluation in 93 Caucasian women with obesity (OB) and non-obesity (NOB).

**Results:**

In OB, imbalance in metabolism of carbohydrate and lipid, autonomic nerve activity, and secreted factors were confirmed. In the skin properties in OB, surface roughness was higher by 70%, the water content was lower by 12%, and changes in the lipid profile of stratum corneum ceramide were observed; in particular, a 7% reduction of [NP]-type ceramide, compared with NOB. Moreover, significant redness accompanied by a 34% increase in skin blood flow was observed in OB. Correlation analysis elucidated that the water content was strongly correlated with local skin indices, such as the ceramide composition, redness, blood flow, and TNFα in the stratum corneum, whereas roughness was correlated with the systemic indices, such as serum insulin, leptin, and IL-6.

**Conclusions:**

Characteristics of obesity-associated skin were (A) reduction of the barrier and moisturizing function accompanied by intercellular lipid imbalance, (B) increased redness accompanied by hemodynamic changes, and (C) surface roughness. It was suggested that each symptom is due to different causes in local and/or systemic physiological impairment related to the autonomic nerve-vascular system, inflammation and insulin resistance.

**Electronic supplementary material:**

The online version of this article (10.1186/s12944-017-0608-1) contains supplementary material, which is available to authorized users.

## Backgrounds

Adipose tissue is a unique multifunctional organ serving not only as a simple storage of excess energy, but also as connective tissue, a metabolic organ, endocrine organ, and source of stem cells. Obesity represents adipocyte hypertrophy or hyperplasia, and it has been considered a serious worldwide pandemic. Recent studies demonstrated that excessive accumulation of intra-abdominal fat, termed “visceral fat”, plays an important role in the complex cascade of metabolic syndrome and diabetes. Increased visceral fat secretes various hormones, fatty acids and pro-inflammatory cytokines, such as IL-6 and TNF-a, termed “adipokines”, and they induce insulin resistance in distant organs such as the liver, skeletal muscles, and blood vessels [1–3].

Clinical observations of skin disorders in patients with obesity have been reported including pressure ulcers, lymphedema, psoriasis, nigricum, cellulite, and striae [4, 5]. Intractable pathological changes in the skin also occur in diabetes patients, which are due to impairment of the circulatory, nervous, and immune systems [6, 7]. We predict that obesity causes impairment of the skin physiology, but obesity-associated changes in skin function and the molecular mechanisms have not been sufficiently clarified. The objective of this study was to characterize the skin properties of American women with obesity by instrumental measurement, new lipidomic analyses, and biochemical evaluation, and therefore clarify the association between skin and health functions.

## Methods

### Subjects

Caucasian American females, aged between 30 and 49 years, were separated into two groups, the non-obesity group (NOB, *n* = 46) and obesity group (OB, *n* = 47), according to body-mass index (BMI). BMI was calculated from measured value of height and body weight, and the criteria of BMI between NOB and OB was above or below 30 kg/m^2^. All subjects were non-smokers, pre-menopausal, not pregnant, and not on any medications for obesity, diabetes or skin diseases.

### Measurements of body composition and blood pressure

Body weight and body fat were measured by bioelectrical impedance analysis using a body composition meter EW-FA71 (Panasonic, Osaka, Japan). Height was measured by a height rod. Circumferences of hip, and waist at the navel level in the standing position were measured and used for calculation of waist/hip ratio (WHR). Blood pressure was measured using a digital blood pressure monitor (ReliOn HEM-780REL, Omron Healthcare, Inc., IL, USA).

### Blood analysis

Blood sampling was performed after fasting for 12 h. The target biochemical markers and hormones in the serum samples were determined by enzyme immunoassay or radio immunoassay according to the manufacturer’s instruction (Additional file 1: Table S1). The target biochemical markers were glucose, hemoglobin A1c (HbA1c), triglyceride (TG), total cholesterol (Total-Cho), low-density lipoprotein cholesterol (LDL-Cho), high-density lipoprotein cholesterol (HDL-Cho), c-reactive protein (CRP), insulin, leptin, adiponectin, and IL-6. Homeostasis model assessment-insulin resistance (HOMA-IR) was calculated from the fasting level of insulin and glucose. We identified MetS as an index of metabolic syndrome grade. The number of items that met the criteria [8] was counted (from 0 to 5 points) as the MetS (Additional file 1: Table S1).

### Analysis of autonomic nerve activity

For the characterization of autonomic nerve activity, electrocardiogram during sleeping hours was obtained using a heart rate monitor (ActiHeart; CamNtech Inc., TX, USA) with 1mS resolution. The data were transferred to the Actiheart software (CamNtech Inc.), and heart rate variability and inter-beat interval were analyzed. The heart rate (HR), the low frequency spectrum (LF); (0.05–0.15 Hz), the high frequency spectrum (HF); (0.15–0.4 Hz), and total power (TF) for 4 h (2–6 h after asleep) were calculated. HF and LF/HF were adopted as indices of parasympathetic nerve activity and sympathetic nerve activity, respectively [9]. TF was adopted as the total activity of autonomic nerves.

### Instrumental measurements

Subjects rested for over 20 min for stabilization after face cleansing. To evaluate the skin attributes, facial images in the sitting position were obtained with the Visia-CR2 imaging system (Canfield Scientific, Inc., NJ, USA). The following instrumental measurements were taken with the region of interest (ROI) in the right cheek. Measurements of capacitance and trans-epidermal water loss (TEWL), as indices of water content and barrier function of the stratum corneum (SC) in the epidermis, using Corneometer-Tewameter MPA 580 (Courage + Khazaka electronic GmbH, Cologne, Germany), surface structure, such as roughness, scaliness, and wrinkles, using Visioscan VC98 (Courage + Khazaka electronic GmbH), and skin tone using a colorimeter CR-400 (Konica Minolta, Inc., Tokyo, Japan) were performed according to the manufacturers’ instruction. Color difference was calculated from obtained parameters, a*, b* and L* [10] (Additional file 1: Table S1). Redness was scored from 1 to 5 points on the forehead, nose, upper cheek and lower cheek using images obtained with Visia-CR2. The total score of these four regions was adopted as the individual redness score.

To evaluate the microcirculation in the skin, including subcutaneous tissue, we adopted two measurements. One was the laser speckle blood flow system, PeriCam PSI NR (Perimed AB, Järfälla, Sweden), which has high sensitivity to detect superficial blood circulation by real-time imaging. Continuous images of ROI were obtained during 20 s, and analyzed using the software PIMSoft (Perimed AB) to calculate average values and standard deviations of perfusion in the ROI. Tissue-blood oxygenation monitor, BOM-L1TRSF (Omegawave Inc., Tokyo, Japan) based on near infrared spectroscopy (NIRS), was used to quantify oxygenated hemoglobin (Oxy-Hb), deoxygenated hemoglobin (Deoxy-Hb), and total hemoglobin (Total-Hb) in subcutaneous tissues at a depth 3–5 mm. To measure the core and skin surface temperature, an ear thermometer (ThermoScan 5; Braun GmbH, Kronberg, Germany) and skin thermometer (Thermo-meter ST500, Courage + Khazaka electronic GmbH) were used.

### Lipidomic analysis of intercellular lipid

After the instrumental measurements, SC specimens in the ROI were collected by tape stripping 3 times. Lipids were extracted from these specimens and analyzed as described in Additional file 1: Table S1 and detailed in a previous report [11]. Lipid profile for straight-chain fatty acids (FFAs), branched-chain FFAs, mono-unsaturated fatty acids, ceramides (Cers), cholesterol, and cholesterol sulfate were analyzed. Cers includes eleven types: Cer [NDS], [NS], [NH], [NP], [ADS], [AS], [AH], [AP], [EOS], [EOH] and [EOP]. Cer [NDS] contains non-OH fatty acids [N] and dihydrosphingosines [DS], Cer [NS] contains [N] and sphingosines [S], Cer [NH] contains [N] and 6-hydroxy sphingosines [H], Cer [NP] contains [N] and phytosphingosines [P], Cer [ADS] contains α-OH fatty acids [A] and [DS], Cer [AS] contains [A] and [S], Cer [AH] contains [A] and [H], Cer [AP] contains [A] and [P], Cer [EODS] contains esterified ω-hydroxy fatty acids [EO] and [DS], Cer [EOS] contains [EO] and [S], Cer [EOH] contains [EO] and [H], and Cer [EOP] contains [EO] and [P]. Protein concentration was quantified using BCA assay kit (Thermo Scientific, IL, USA).

### Cytokines in the stratum corneum

SC specimens were extracted with PBS with 0.1% Triton-X 100 (Sigma-Aldrich Co., Llc., MO, USA) for two hours, and centrifuged for 15 min at 2000 ×g. Obtained supernatants were used for quantification of protein and cytokines. Protein concentration was quantified using a BCA assay kit (Thermo Scientific). Concentration of IL-1α, IL-1 receptor antagonist (IL-1ra), and TNFα were determined using ELISA (R&D systems, Inc., MN, USA), and normalized by the protein concentration. The [IL-1ra / IL-1α] ratio was calculated as a dermal inflammatory index [12].

### Statistic

Distribution of data was presented by box-plotting, showing mean value, 25th percentile, 50th percentile, and 75th percentile of the group. Difference of mean values was compared between the two groups using an unpaired Student’s t-test or Mann-Whitney’s U-test. *p* < 0.05 was taken as an indicator of significance. Correlation was tested by Pearson’s method for parametric analysis or Spearman’s rank-method for nonparametric analysis, and a multiple linear regression analysis was performed using GraphPad Prism6 (GraphPad Software, Inc., CA, USA).

## Results

### Physical profile, and glucose and lipid metabolic functions

The physical indices of NOB and OB are shown in Table 1 and Additional file 2: Figure S1. There was no significant difference in the age between the 2 groups, but the mean BMI, body fat, waist and hip circumferences in OB were 1.58- and 1.77-times higher/larger than those in NOB, showing marked fat accumulation. WHR in OB was significantly higher than that in NOB and the mean exceeded 0.90. Both systolic and diastolic blood pressure were significant higher in OB than that in NOB. Regarding the lipid metabolic system, the mean TG was 150 mg/dL or higher in OB, which was significantly higher. No significant difference was noted in Total-Cho between the 2 groups, but LDL-Cho was significantly higher in OB. Inversely, the mean HDL-Cho was 50 mg/dL in OB, which was significantly lower. Regarding the glucose metabolic system, glucose, insulin, HbA1c, and HOMA-IR were significantly higher or tended to be higher in OB than in NOB, and MetS was also markedly higher in OB than in NOB.

**Table 1:** Profile of body composition and markers in serum and stratum corneum

	NOB-group	OB-group	OB/NOB fold	*p*-value
Age (year-old)	40.6 ± 0.8	39.2 ± 0.9	0.97	N.S.
BMI (Kg/m^2^)	22.5 ± 0.3	35.5 ± 0.5	1.58	<0.0001
Body fat (%)	29.7 ± 0.6	52.5 ± 1.1	1.77	<0.0001
WHR	0.85 ± 0.01	0.92 ± 0.01	1.08	<0.0001
Systolic Blood Pressure	115.8 ± 2.8	124.7 ± 2.3	1.08	<0.05
Diastolic Blood Pressure	71.4 ± 1.6	80.4 ± 1.5	1.13	<0.001
TG (mg/dL)	80.6 ± 6.2	151.3 ± 15.2	1.88	<0.001
Total-Cho (mg/dL)	195.6 ± 5.0	200.9 ± 4.6	1.03	N.S.
LDL-Cho (mg/dL)	106.4 ± 4.4	118.7 ± 4.0	1.12	<0.05
HDL-Cho (mg/dL)	69.6 ± 2.7	49.5 ± 2.4	0.71	<0.0001
Glucose (mg/dL)	85.2 ± 1.1	95.5 ± 5.1	1.12	<0.10
Insulin (mU/mL)	5.28 ± 0.34	12.10 ± 1.10	2.29	<0.0001
HbA1c (%)	5.39 ± 0.03	5.741 ± 0.12	1.07	<0.01
HOMA-IR	1.12 ± 0.08	3.04 ± 0.42	2.70	<0.001
MetS (point)	0.55 ± 0.10	1.67 ± 0.15	3.02	<0.001
Leptin (ng/mL)	16.92 ± 1.48	55.42 ± 2.76	3.28	<0.0001
Adiponectin (mg/mL)	16.72 ± 1.34	10.56 ± 0.88	0.63	<0.001
IL-6 (pg/mL)	1.06 ± 0.12	3.39 ± 0.72	3.19	<0.01
CRP (mg/dL)	0.24 ± 0.06	0.89 ± 0.14	3.68	<0.0001
SC IL-1ra/IL-1α	96.5 ± 14.0	130.7 ± 22.0	1.36	N.S.
SC TNFα (pg/L)	24.8 ± 3.08	25.9 ± 3.81	1.04	N.S.

Indices in body composition, glucose metabolism, lipid metabolism, secreted markers and inflammatory markers in serum and stratum corneum (SC) were presented as the mean ± S.E.M. The ratio of values in the obesity group to the non-obesity group (OB/NOB fold) and statistical significance were presented in each column

### Autonomic nerve activity

No marked difference was observed in the sympathetic nerve activity index LF/HF between the 2 groups, but the parasympathetic nerve activity index HF in OB was approximately 59% of that in NOB, which was significantly lower, and the total autonomic nerve activity index TF was also low (54%), demonstrating marked reduction of autonomic nerve activity in OB. Heart rate (HR) was higher by approximately 9 (beats/min) in OB than in NOB (Additional file 2: Figure S2). Abnormal changes in the autonomic nervous and circulatory systems in OB were demonstrated.

### Skin function

**Fig. 1:**
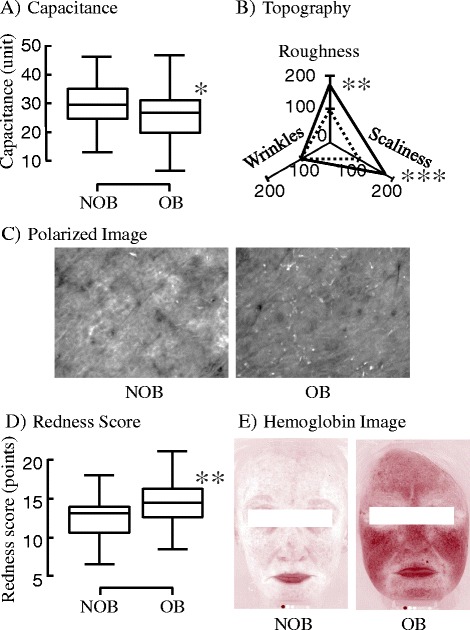
Analyses of epidermal function and redness. Capacitance as a parameter for water content in stratum corneum was measured by Corneometer. Values in the non-obesity group (NOB) and the obesity (OB) were presented by box-plot with 75th percentile (upper side), median (inner line), 25th percentile (lower side) and whisker lines (upper and bottom line) (**a**). Topography of skin surface structure was evaluated by VisioScan. Values for each parameter in the NOB and the OB were presented by radar graph with solid line and dashed line, respectively (**b**). Typical polarized light images acquired by VisioScan were shown (**c**). Facial images were acquired by Visia-CR and analyzed to evaluate redness (**d**). Typical hemoglobin images obtained by Visioscan were shown (**e**). *: *p* < 0.05, **: *p* < 0.01, ***: *p* < 0.001, compared between mean values in two groups

The main functional indices of the epidermis and SC, skin surface profile and skin tone are shown in Fig. 1 and Additional file 1: Table S2. TEWL tended to be higher in OB than in NOB, and the capacitance was approximately 12% lower in OB than in NOB, demonstrating a significant difference between the groups (Fig. 1a). The surface profile markedly differed between the 2 groups: the grades of roughness and scaliness were higher by 70 and 87% in OB than in NOB, respectively (Fig. 1b). The typical polarized light images of the skin surface showed rough texture and desquamation in OB (Fig. 1c). It was observed that the barrier and moisturizing functions decreased with obesity, and the skin was markedly dried and rough. In the comparison of skin tone, the b* value was significantly lower, and the a* value tended to be higher in OB than that in NOB. No significant difference was observed in L* between the groups. Tendency of increase in redness and decrease in yellowish with obesity were noted. ΔE*, the color difference, between the 2 groups was 2.06, and this corresponds to the “Noticeable” level in the official criteria [9]. The mean redness score in OB was higher by 0.81 points than that in NOB, which was significantly higher (Fig. 1d). As shown in typical hemoglobin images acquired using VisiaCR (Fig. 1e), marked redness was observed in OB, and redness was particularly marked in the upper cheek.

### Microcirculation

**Fig. 2:**
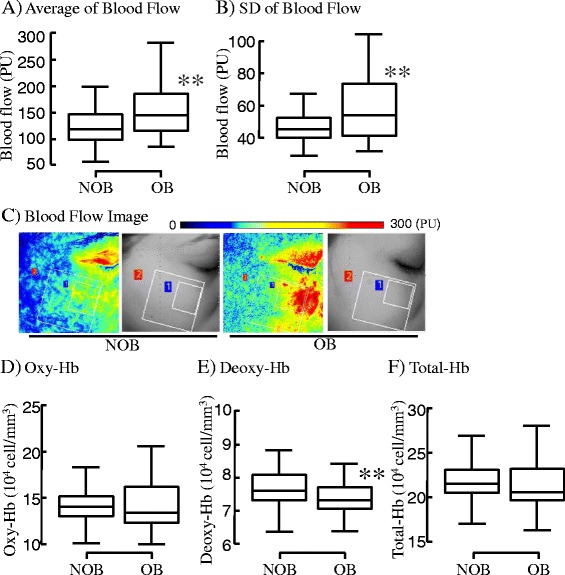
Analyses of blood flow and hemoglobin dynamics. Real-time blood flow images were obtained by a laser speckle blood flow system and analyzed to characterize perfusion in the skin. Average and standard deviation (SD) of blood flow in the region of interest (ROI) were presented (**a** and **b**, respectively). Typical blood flow images were shown (**c**). Hemoglobin dynamics in the subcutaneous tissue was evaluated by a tissue-blood oxygenation monitor to analyze Oxy-Hb (**d**), Deoxy-Hb (**e**) and Total-Hb (**f**) **: *p* < 0.01, compared between mean values in two groups

The properties of blood flow in ROI in the cheek are shown in Fig. 2a, b, and other results are summarized in Additional file 1: Table S3. The average and standard deviation (SD) of blood flow in OB was significantly higher/larger by 1.34- and 1.25-times than that in NOB, showing uneven increase of blood flow. As shown in the blood flow images (Fig. 2c), regions with a high blood flow were distributed in the upper cheek region in OB. No significant difference was observed in the core body temperature between the 2 groups, but the skin temperature tended to be higher in OB than in NOB, and the difference in the mean was 0.43 °C.

On analysis of hemoglobin dynamics in subcutaneous tissue using NIRS (Fig. 2d, e, f), no significant difference was observed in Oxy-Hb or Total-Hb between the groups, but a significant difference was observed in Deoxy-Hb, and it was lower by 0.33×10^4^ cell/mm^3^, i.e., approximately 4%, in OB than in NOB.

### Secretory factors and inflammatory markers

Regarding the blood levels of adipose tissue-derived hormones in OB, the leptin level markedly rose, and in contrast, the level of the anti-inflammatory hormone, adiponectin, decreased to approximately 63% (Table 1, Additional file 2: Figure S3). The levels of IL-6, which is an adipose tissue-derived proinflammatory cytokine, and the systemic inflammatory marker, CRP, were significantly higher in OB by 3-times or more than those in NOB. In addition, the mean levels of skin inflammatory markers, the IL-1ra/IL-1α ratio [12], and TNFα in the SC, were approximately 1.35- and 1.04-times higher in OB than in NOB, respectively, but the differences were not significant because variations among individuals were large.

### Intercellular lipids

In the profiling of intercellular lipids in SC, no significant difference was observed in the total amount of Cers, cholesterol, fatty acids, or cholesterol sulfate composing intercellular lipids between the 2 groups (Additional file 1: Table S4). The fatty acid components: linear/branched fatty acid and monounsaturated fatty acid, had no quantitative difference between the 2 groups (data not shown).

**Fig. 3:**
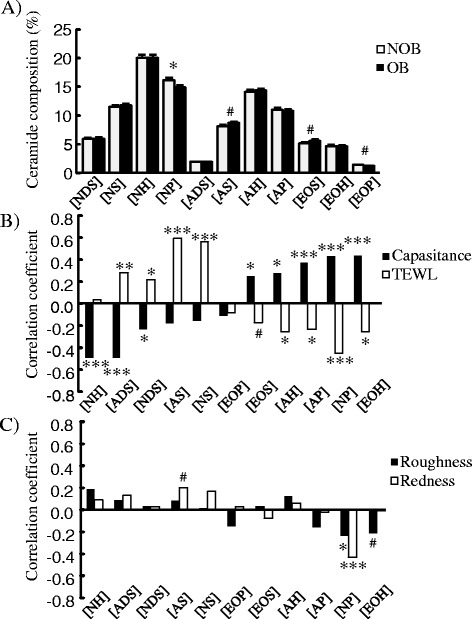
Profile of stratum corneum ceramides and correlation with skin attributes. Structural profiling of ceramides was performed by lipidome, and values for eleven types in the NOB and the OB were presented as the mean ± S.E.M (*a*). #: *p* < 0.10, *: *p* < 0.05, compared between mean values in two groups. Correlation of ceramide composition with capacitance, TEWL, roughness and redness was analyzed, and correlation efficient was shown (**b**, **c**). #: *p* < 0.10, *: *p* < 0.05, **: *p* < 0.01, ***: *p* < 0.001

In detailed profiling of Cers by the molecular species, quantitative and qualitative differences were observed between the 2 groups (Fig. 3a). The composition ratio of Cer [NP] in ceramide in OB was significantly lower by approximately 7% than that in NOB. In the distribution of the Cer [NP] chain length was compared, the amount of long-chain elements (C45-C48) was reduced in OB (Additional file 2: Figure S4).

### Analysis of correlation

Focusing on reductions of the moisturizing and barrier functions, and increases in skin surface roughness and redness accompanying obesity, the correlation with each parameter in all subjects was described in this section. The indices correlated with capacitance were TEWL, roughness, scaliness, the average and SD of blood flow, redness, Oxy-Hb, Total-Hb, L*, and TNFα in the SC. All these were local indices, and the correlation coefficient was within a range of approximately ±0.22 to ±0.39 (Table 2).

**Table 2:** Indices correlated with capacitance

	L/S	Correlation efficiency	*p*-value
TEWL	L	−0.437	<0.001
Average of Blood Flow	L	−0.388	<0.001
Roughness	L	−0.315	<0.01
Scaliness	L	−0.303	<0.01
SD of Blood Flow	L	−0.280	<0.05
Redness Score	L	−0.280	<0.05
Oxy-Hb	L	−0.268	<0.05
SC TNFα	L	−0.263	<0.05
Skin Tone L*	L	0.230	<0.05
Total-Hb	L	−0.220	<0.05

Correlation with capacitance was analyzed, and indices with significant correlation were listed in the table. Local indices (L) or systemic indices (S) were shown in L/S column. a* and L* are color property parameters.

The redness score exhibited strong positive correlations with a*, and the average and SD of blood flow, and a strong inverse correlation with L* (Table 3). It was also correlated with the capacitance, TEWL, and scaliness, as well as with systemic indices. Positive correlations of the redness with the body fat, BMI, blood pressure, and autonomic nerve activity were observed. Serum TG, insulin, Total-Cho, and IL-6 also showed weak correlation with redness score.

**Table 3:** Indices correlated with redness

	L/S	Correlation efficiency	*p*-value
Skin Tone L*	L	−0.578	<0.001
Skin Tone a*	L	0.517	<0.001
Systolic Blood Pressure	S	0.339	<0.01
Average of Blood Flow	L	0.332	<0.01
Body Fat	S	0.300	<0.01
Subcutaneous Fat	L	0.293	<0.01
BMI	S	0.290	<0.01
SD of Blood Flow	L	0.282	<0.05
Capacitance	L	−0.280	<0.05
TEWL	L	0.247	<0.05
TG	S	0.243	<0.05
Insulin	S	0.242	<0.05
Autonomic Nerve TF	S	−0.242	<0.05
Autonomic Nerve HF	S	−0.242	<0.05
Scaliness	L	0.232	<0.05
Autonomic Nerve LF/HF	S	0.231	<0.05
Total-Cho	S	0.231	<0.05
IL-6	S	0.226	<0.05

Correlation with redness was analyzed, and indices with significant correlation were listed in the table. Correlation efficient was presented. Local indices (L) or systemic indices (S) were shown in L/S column. a* and L* are color property parameters

Skin surface roughness was inversely correlated with the above capacitance, positively correlated with scaliness and the mean blood flow, and correlated with many systemic indices (Table 4). Specifically, it was strongly correlated with BMI, body fat, leptin, insulin, IL-6 (correlation coefficient: 0.4 or higher), and CRP, and inversely correlated with HDL-Cho and adiponectin. It was also weakly correlated with blood pressure and autonomic nerve activity.

**Table 4:** Indices correlated with roughness

	L/S	Correlation efficiency	*p*-value
Leptin	S	0.585	<0.001
BMI	S	0.561	<0.001
Body fat	S	0.549	<0.001
Scaliness	L	0.503	<0.001
Insulin	S	0.466	<0.001
IL-6	S	0.463	<0.001
MetS	S	0.373	<0.001
HDL-Cho	S	−0.346	<0.01
HOMA-IR	S	0.317	<0.01
Capacitance	L	−0.315	<0.01
Diastolic Blood Pressure	S	0.287	<0.01
Autonomic nerve LF/HF	S	0.278	<0.01
Autonomic nerve TF	S	−0.274	<0.05
Autonomic nerve HF	S	−0.261	<0.05
Systolic Blood Pressure	S	0.251	<0.05
Average of Blood Flow	S	0.244	<0.05
CRP	S	0.237	<0.05
Skin Tone b*	L	−0.226	<0.05
Adiponectin	S	−0.223	<0.05

Correlation with roughness was analyzed, and indices with significant correlation were listed in the table. Correlation efficient was presented. Local indices (L) or systemic indices (S) were shown in L/S column. b* is a color property parameters

Furthermore, correlation between CS Cers composition and skin properties are tested (Fig. 3b, c). Cer [NP] showed marked correlation with the capacitance and TEWL positively and inversely, respectively. Other Cers such as [EOH] and [AP] were positively, and [NH] and [ADS] were inversely correlated with SC function. On the other hand, the skin surface roughness and redness score lacked correlations with Cers, but both indices demonstrated inverse correlations only with Cer [NP].

**Fig. 4:**
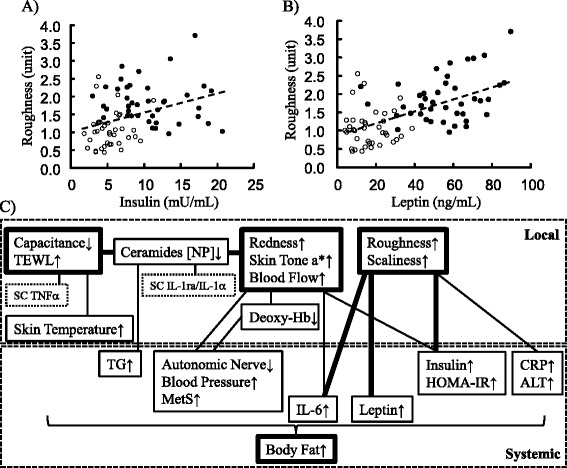
Obesity-associated changes of skin property and correlation between indices. Correlation of skin surface roughness with serum insulin and leptin was represented by scatter plots (**a**, **b**). Open circles show NOB, and filled circles show OB. Changes of local and systemic indices associated with obesity (↑: increase, ↓: decrease) and correlation were summarized in a diagram (**c**). Solid lines represent statistically significant correlation between indices, and thick lines represent strong correlation (correlation efficiency >0.40 or < −0.4)

A schematic drawing of correlation between representative indices and typical correlation graphs were shown in Fig. 4. Capacitance – redness axis showed independent relationship of roughness – systemic parameters axis. Focusing on the relation between the capacitance and its related factors, a multiple linear regression was calculated to predict capacitance based on TEWL, Blood Flow, Cer [NP], SC TNFα, SC IL-1ra/IL-1α, and Deoxy-Hb in local parameters, and serum Leptin, serum IL-6, serum Insulin, Systolic Blood Pressure, and TF of autonomic nerve in systemic parameters. A significant regression equation was found (F (11, 45) = 3.1205, *p* < 0.01), with an adj R^2^ of 0.294. Predicted capacitance is equal to 56.330–0.040 (Blood Flow) - 0.416 (TEWL) - 0.096 (Leptin) - 0.061 (Blood pressure) - 1.817 (Deoxy-Hb) - 19.739 (TNFα) - 0.235 (IL-6) + 0.000 (TF) + 0.300 (Cer [NP]) + 0.217 (Insulin) + 0.027 (IL-1ra/IL-1α). Highly significant predictors of the capacitance were not identified, but Blood Flow (*p* = 0.047, *t* = −2.039) and TEWL (*p* = 0.074, *t* = −1.828) were top candidates.

## Discussion

Visceral adiposity induces hyperinsulinemia, hyperglycemia, and hyperlipidemia, and causes systemic insulin resistance, although elucidation of this mechanism has been progressing [1–3, 13–15]. Association of excessive accumulation of subcutaneous fat with breast cancer and lymphedema has also been reported [15, 16]. The chronic inflammatory skin disease psoriasis was associated with type 2 diabetes, body mass index and obesity in a study of Danish twins, and the study also suggests the possibility of a common cause or relationship between psoriasis and obesity [17]. Moreover, diabetes induces severe nerve and vascular disorders, bacterial infection, ulcer, retinopathy [18]. We clarified the properties and functional characteristics of the skin in women with obesity by objective evaluation and measurement to acquire fundamental knowledge on the association between obesity and skin function.

In the WHO definition, ‘a BMI of 25 or higher is regarded as overweight and 30 or higher is regarded as obesity’. In the criteria concerning metabolic syndrome, in addition to the specifications concerning blood pressure and blood factors, ‘a waist circumference of 88 cm or larger and WHR of 0.85 or higher’ are proposed as the rough standards indicating an increase in disease risks [8]. Patients with obesity and diabetes being treated were not included in this study, but the means exceeded the above criteria in OB, and almost indices were consistent with or close to the criteria. And reduction of total autonomic nerve activity due to reduction of parasympathetic nerve activity, increases in the heart rate and blood pressure were observed in OB, as in previous reports [19–21], which showed relative dominance of sympathetic nerve activity. In addition, high levels of the blood inflammatory markers observed in OB indicate progression of systemic inflammation-like changes.

In the obesity described above, characteristic changes were noted in the skin properties, dryness, roughness, and characteristic skin tone. Lesions accompanied by over-drying, such as hyperkeratosis and psoriasis, was reported as skin diseases in patients with obesity [4, 5, 22]. In our study, severe dryness in women with obesity was quantitatively observed, suggesting dysfunction of SC.

Intercellular lipids such as SC Cers play a critical role in epidermal moisturizing and barrier functions [23, 24]. We performed comprehensive lipid analysis using lipidomes [11], characterized intercellular lipids accompanied with obesity, and initially found differences in the Cer profile. In atopic dry skin, decreases of Cer [NP] and [NH] in sphingoids with non-hydroxy fatty acid, and [EOS], [EOH], and [EOP] in sphingoids with esterified ϖ-hydroxy fatty acid were observed [25]. In healthy subjects, Cers described above and other Cers, such as [NS], [NDS], [AS], and [AP], decreased as the barrier function decreased [26]. In our study, the epidermal water content was strongly correlated with many molecular species of Cers consistent with the above previously reported findings. In particular, quantitative and qualitative changes in Cer [NP], which is a phytosphingosine with non-hydroxy fatty acid, may sensitively reflect several obesity-associated changes in SC function. We also found specific ceramide species, Cer [NH] and [ADS], inversely correlated only with TEWL. The physiological roles of these characteristic molecular species in modulation of the epidermal metabolism have not been elucidated.

Another characteristic change observed with obesity is change of skin color properties, particularly an increase in cheek redness and a significant decrease in b*. It is considered that redness and a* depend on the blood hemoglobin level, whereas b* is mainly markedly influenced by coloring substances, such as melanin, glycated protein, and carotene [27]. The IL-1ra/IL-1α ratio in SC has been reported to increase in light-exposed skin and ultraviolet light-induced inflammation [28]. This ratio, blood flow, and skin temperature were high in addition to redness in OB, suggesting that these are mild and chronic skin inflammation-like signs accompanied by local vascular dilatation. The obesity–associated decrease in Deoxy-Hb, without increase of oxygen supply, in subcutaneous tissue was found as a characteristic change in hemoglobin dynamics. This suggests reduced state of energy consumption, such as inflammation or metabolic impairment, and a similar phenomenon is observed in muscle tissue under artificial psychological stress loading [29, 30].

By analyzing the correlations following analysis in all subjects, the indices of local skin and systemic functions correlated with the skin characteristics could be identified, as shown in Fig. 4. The moisturizing and barrier function described suggested to be strongly influenced by not only several molecular species of Cer, but also excessive blood flow, inflammatory changes in skin-subcutaneous tissue and serum IL-6. In previous reports, atopic dermatitis-like spongy changes and reduction of ceramide production induced by cytokines such as IL-1α and TNFα in epidermal cells were observed [31–35]. The following mechanism has been reported that infiltrating macrophages are mobilized by chemotactic factors secreted by enlarged adipocytes in obesity, and secrete pro-inflammatory cytokines through the toll-like receptor-NF-κB pathway [14]. We predict chronic inflammatory changes occur in the skin and/or adipose tissue and it negatively affects the metabolic pathway of intercellular lipids. Increased redness may be caused by not only inflammation but also impaired vascular regulation by autonomic nerve. Multiple regression analysis showed a possibility of microcirculation as a candidate factor with large impact on these functional alterations of SC.

On the other hand, it was suggested that the skin surface roughness and scaliness are strongly influenced by systemic factors such as IL-6 and leptin, so-called “adipokines” and insulin, suggesting roughening is exaggerated by systemic inflammation and systemic/dermal insulin resistance in a complex manner. Interaction between keratinocytes, fibroblasts and adipokines might be related to this phenomenon [36]. Elucidation of the main cause and mechanism is awaited. In a recent study, “obesity paradox” in skin function was described [37]. It showed lower TEWL and higher SC hydration of forehead and zygomatic area in overweight group (BMI:25–29.9) and obesity I-II group (BMI:30–39.9) than normal weight and morbidly obesity group. In our analysis, a similar tendency of TEWL and capacitance were not observed both in cheek area of overweight grade subjects in NOB and that of obesity I-II grade subjects in OB (data not shown). TEWL values in our study were higher than that reported in the above study, suggesting differences in anatomical area, ages, living environmental condition including season and life style habit. Further cohort study or comprehensive investigations for more wide range of BMI, anatomical areas, and environmental condition are necessary to understand the characteristics of local skin property and its relation to systemic regulatory system.

## Conclusions

We initially characterized the skin properties accompanied with obesity, and demonstrated that obesity is an aggravating factor of skin properties. It was suggested that reduction of the moisturizing and barrier functions of the SC is strongly associated with changes in local skin function, such as specific changes in the amount and composition of Cers, excessive blood flow, and inflammation-like symptom including redness, which is correlated with imbalanced systemic function. Independently of the skin barrier – redness axis, roughness is strongly associated with blood factors, such as pro-inflammatory adipokines and insulin. These findings may be important basic knowledge to understand skin dysfunction occurring in obesity and diabetes, and to establish measures to predict, prevent and improve the reduction of quality of life caused by lifestyle disease.

## Additional files

Additional file 1: Table S1. Additional information of materials and methods. **Table S2.** Skin function. **Table S3.** Microcirculation and hemodynamics. **Table S4.** Quantity of intracellular lipid in stratum corneum. (DOCX 27 kb)

Additional file 2: Figure S1. Body fat and systemic metabolism. **Figure S2.** Autonomic nerve activity. **Figure S3.** Adipokines and inflammatory markers. **Figure S4.** Profile of ceramide [NP] (PDF 248 kb)

## Abbreviations

TNFα: Tumor-necrotic factor alpha
BMI: Body mass index
Cer: Ceramide
CRP: C-reactive protein
Deoxy-Hb: deoxy-hemoglobin
FFA: Free fatty acid
HDL-Cho: high-density lipoprotein cholesterol
HF: High frequency spectrum
HOMA-IR: Homeostasis model assessment-insulin resistance
HR: Heart rate
IL: Interleukin
LDL-Cho: low-density lipoprotein cholesterol
LF: Low frequency spectrum
NIRS: Near infrared spectroscopy
NOB: Non-obesity group
OB: Obesity group
Oxy-Hb: Oxy-hemoglobin
ROI: Region of the interest
SC: Stratum corneum
SD: Standard deviation
TEWL: Trans-epidermal water loss
TF: Total power of frequency spectrum
TG: Triglyceride
Total-Cho: Total cholesterol
Total-Hb: Total hemoglobin
WHO: World health organization
WHR: Waist/hip ratio
