# Correction to: Characterization of skin function associated with obesity and specific correlation to local/systemic parameters in American women

**DOI:** 10.1186/s12944-017-0632-1

**Published:** 2017-12-06

**Authors:** Shinobu Mori, Akiko Shiraishi, Karen Epplen, Desiree Butcher, Daiki Murase, Yuka Yasuda, Takatoshi Murase

**Affiliations:** 1 Biological Science Laboratories, Kao Corporation, 2606 Akabane, Ichikai-machi, Haga-gun, Tochigi, 321-3497 Japan; 2 Spring Grove Laboratories, 375 Thomas More Parkway, Suite 112, Crestview Hills, KY 41017 USA; 3 Analysis Science Laboratories, Kao Corporation, 2606 Akabane, Ichikai-machi, Haga-gun, Tochigi, 321-3497 Japan

## Correction

Following publication of the original article [1], the authors identified an error. In the description in Fig. 1b the “solid line” ←→ “dashed line” should be exchanged.

The original article has been updated.
