# Supplementary material for: Characterization of skin function associated with obesity and specific correlation to local/systemic parameters in American women
Source: Lipids Health Dis. 2017 Nov 13;16:214. doi: 10.1186/s12944-017-0608-1 (PMC5683228; doi:10.1186/s12944-017-0608-1)
Supplement: Supplementary file 1 — Additional information of materials and methods. Table S2. Skin function. Table S3. Microcirculation and hemodynamics. Table S4. Quantity of intracellular lipid in stratum corneum. (DOCX 27 kb) [file 12944_2017_608_MOESM1_ESM.docx]

**Table S1. Additional information of materials and methods**

Supplemental information for each analysis was shown.

| Blood analysis:  Glucose and hemoglobin A1c (HbA1c) were quantified using the Beckman Coulter System AU5800 (Beckman Coulter, Inc., CA, USA). Triglyceride (TG), total cholesterol (Total-Cho), low-density lipoprotein cholesterol (LDL-Cho), high-density lipoprotein cholesterol (HDL-Cho), and c-reactive protein (CRP) were quantified using the assay system of LSI Medience Co., Ltd. (Tokyo, Japan). Insulin, leptin, adiponectin, and IL-6 were quantified using the assay kit from Abbott Japan Co., Ltd. (Tokyo, Japan), Merck Millipore (Darmstadt, Germany), and R&D systems Inc. (MN, USA), respectively. |
| --- |
| Evaluation of MetS:  The World Health Organization (WHO) proposed the following criteria of metabolic syndrome in women [8]; 1) blood glucose: over 110 mg/dL, 2) blood TG: over 150mg/dL, 3) HDL-Cho: under 39 mg/dL, 4) systolic blood pressure and diastolic blood pressure: over 140 mmHg and 90 mmHg, respectively, and 5) WHR: over 0.85. The number of items that met the criteria was counted (from 0 to 5 points) as the MetS. |
| Color difference:  Color difference, ΔE, was calculated as below.  ΔE　= √ [(Δa*)^2^+(Δb*)^2^+(ΔL*)^2^]  Δa*, Δb*, ΔL*: difference between mean values of a* , b* and L* , respectively, in the two groups. |
| Lipidomic analysis of intercellular lipid:  Specimens were analyzed with an Agilent 1100 Series LC/MSD single-quadrupole system equipped with a multi-ion source, ChemStation software, a 1100-well-plate autosampler (Agilent Technologies, CA, USA), and a L-column ODS (2.1 mm i.d. ×150 mm; Chemicals Evaluation and Research Institute, Tokyo, Japan) |

**Table S2. Skin function**

Indices in skin function by biometrical measurements and evaluation were presented as the mean ± S.E.M. The ratio of values in the obesity group to the non-obesity group (OB/NOB fold) and statistical significance were presented in each column.

|  | NOB-group | OB-group | OB/NOB fold | p-value |
| --- | --- | --- | --- | --- |
| TEWL (g/ h⋅m^2^) | 17.6 ± 0.7 | 19.4 ± 0.8 | 1.11 | <0.10 |
| Capacitance (U) | 30.1 ± 1.2 | 26.6 ± 1.3 | 0.88 | <0.05 |
| Roughness (U) | 1.08 ± 0.07 | 1.83 ± 0.09 | 1.70 | <0.0001 |
| Scaliness (U) | 0.49 ± 0.05 | 0.92 ± 0.07 | 1.87 | <0.0001 |
| Wrinkles (U) | 42.2 ± 0.6 | 41.4 ± 0.6 | 0.98 | N.S. |
| Skin Tone a* | 14.6 ± 0.4 | 15.2 ± 0.4 | 1.05 | N.S. |
| Skin Tone b* | 15.4 ± 0.3 | 13.5 ± 0.3 | 0.87 | <0.0001 |
| Skin Tone L* | 62.9 ± 0.4 | 62.8 ± 0.4 | 1.00 | N.S. |
| Redness Score (points) | 10.4 ± 0.3 | 11.2 ± 0.4 | 1.08 | <0.05 |

**Table S3. Microcirculation and hemodynamics**

Indices in perfusion were presented as the mean ± S.E.M. The ratio of values in the OB to the NOB (OB/NOB fold) and statistical significance were presented in each column.

|  | NOB-group | OB-group | OB/NOB fold | p-value |
| --- | --- | --- | --- | --- |
| Average of Blood Flow (PU) | 119.6 ± 5.4 | 159.7 ± 9.3 | 1.34 | <0.001 |
| SD of Blood Flow (PU) | 47.7 ± 2.3 | 59.7 ± 3.2 | 1.25 | <0.01 |
| Core Temperature (°C) | 36.91 ± 0.04 | 36.93 ± 0.05 | 1.00 | N.S. |
| Skin Temperature (°C) | 30.54 ± 0.15 | 30.97 ± 0.19 | 1.01 | <0.10 |
| Oxy-Hb (10^4^ cell/mm^3^) | 13.9 ± 0.3 | 14.0 ± 0.5 | 1.01 | N.S. |
| Deoxy-Hb (10^4^ cell/mm^3^) | 7.67 ± 0.09 | 7.35 ± 0.17 | 0.96 | <0.01 |
| Total-Hb (10^4^ cell/mm^3^) | 21.6 ± 0.4 | 21.4 ± 0.5 | 0.99 | N.S. |

**Table S4. Quantity of intracellular lipid in stratum corneum**

Intercellular lipid of stratum corneum was analyzed by lipidome, and values were presented as the mean ± S.E.M. The ratio of values in the OB to the NOB (OB/NOB fold) and statistical significance were presented in each column.

|  | NOB-group | OB-group | OB/NOB fold | p-value |
| --- | --- | --- | --- | --- |
| Ceramides (ng/μg protein) | 7.53 ± 0.45 | 7.18 ± 0.19 | 0.95 | N.S. |
| Cholesterol (ng/μg protein) | 5.61 ± 0.49 | 6.35 ± 0.58 | 1.13 | N.S. |
| Fatty Acid (ng/μg protein) | 5.13 ± 0.71 | 5.78 ± 0.64 | 1.13 | N.S. |
| Cholesterol Sulphate  (ng/μg protein) | 0.75 ± 0.06 | 0.83 ± 0.07 | 1.12 | N.S. |
