# Supplementary material for: Characterization of skin function associated with obesity and specific correlation to local/systemic parameters in American women
Source: Lipids Health Dis. 2017 Nov 13;16:214. doi: 10.1186/s12944-017-0608-1 (PMC5683228; doi:10.1186/s12944-017-0608-1)
Supplement: Supplementary file 2 — Body fat and systemic metabolism. Figure S2. Autonomic nerve activity. Figure S3. Adipokines and inflammatory markers. Figure S4. Profile of ceramide [NP] (PDF 248 kb) [file 12944_2017_608_MOESM2_ESM.pdf]

Fig.S1

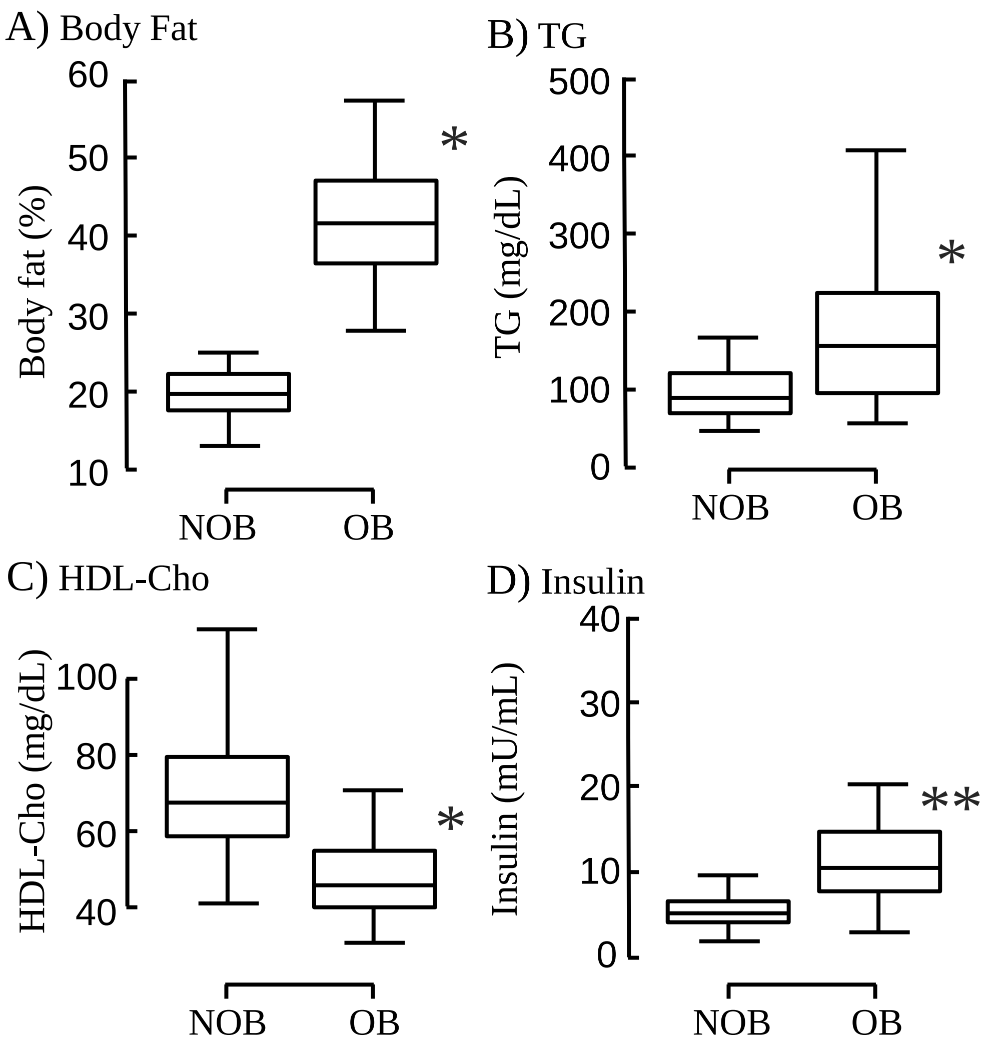

Figure S1. Body fat and systemic metabolism

Values in the non-obesity group (NOB) and the obesity group (OB) were presented by box-plot for body fat (A), serum TG (B), serum HDL-Cho (C) and serum insulin (D). *: $p<0.05$, **: $p<0.01$, compared between mean values in two groups.

Fig.S2

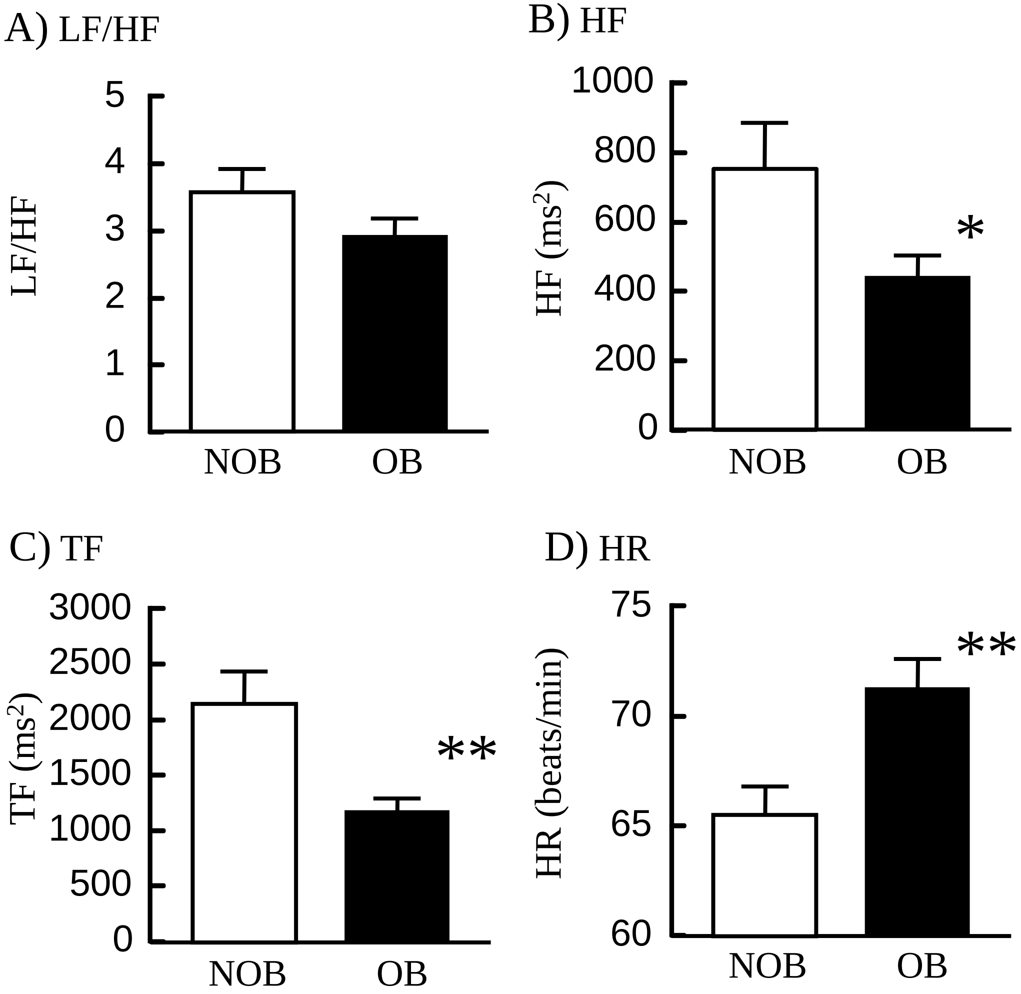

Figure S2. Autonomic nerve activity

Electroradiogram was obtained by a heart rate monitor and analyzed to evaluate autonomic nerve activity. Values in the NOB and the OB were presented as the mean ± S.E.M. for LF/HF as a parameter for sympathetic nerve activity (A), HF as a parameter for parasympathetic nerve activity (B), TF as a parameter for total activity (C) and heart rate (D). *: $p<0.05$, **: $p<0.01$, compared between mean values in two groups.

Fig. S3

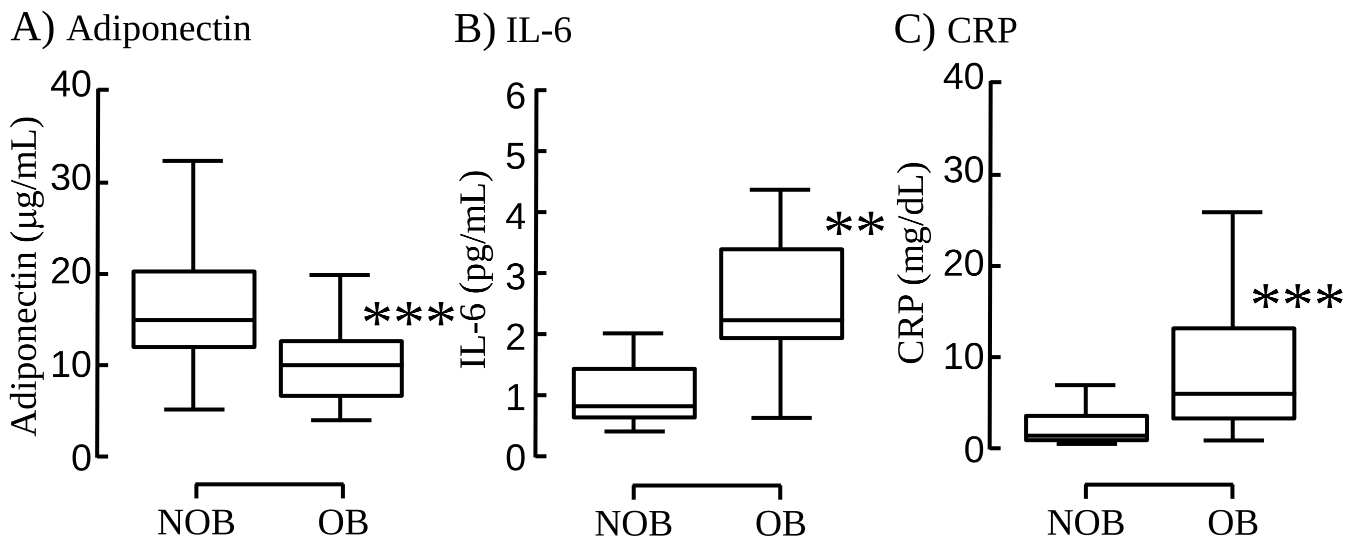

Figure S3. Adipokines and inflammatory markers

Blood analyses were performed by immunoassay, and inflammation-related factors in the NOB and the OB were presented for adiponectin (A), IL-6 (B) and CRP (C) by box-plot. **: $p<0.01$, ***: $p<0.001$, compared between mean values in two groups.

Fig. S4

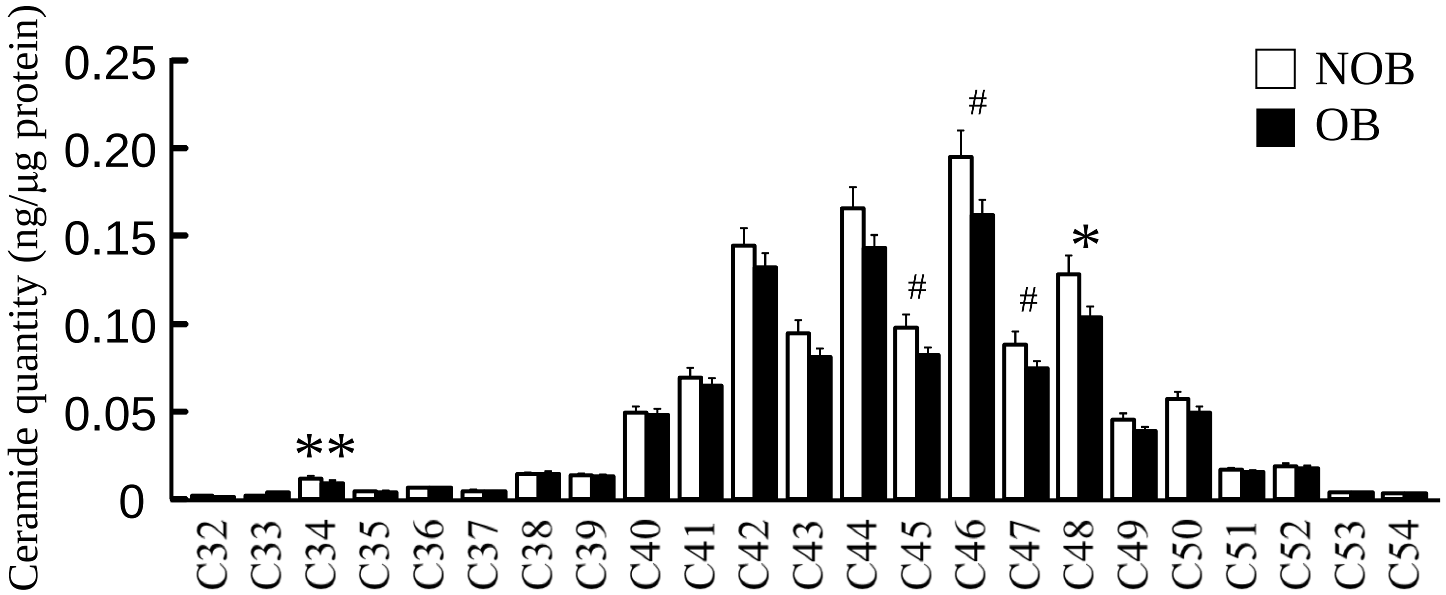

Figure S4. Profile of ceramide [NP]

Profiles of carbon number of ceramide [NP] in the NOB and the OB were presented as the mean ± S.E.M. #: $p<0.10$, *: $p<0.05$, **: $p<0.01$, compared between mean values in two groups.
